# Intra-abdominal rhabdomyosarcoma in a paediatric patient presenting as acute appendicitis

**DOI:** 10.1308/rcsann.2024.0065

**Published:** 2024-07-31

**Authors:** MC Moolamannil, H Khan, S Karim

**Affiliations:** Bedfordshire Hospitals NHS Foundation Trust, UK

**Keywords:** Paediatric surgery, Rhabdomyosarcoma, Acute abdomen

## Abstract

Rhabdomyosarcoma (RMS) is a form of soft tissue sarcoma that can arise from muscle or fibrous tissue almost anywhere in the body. The two major subtypes of RMS are alveolar and embryonal, whereas the two rarer subtypes are pleomorphic, which typically occurs in adults, and the spindle cell/sclerosing variant, typically seen in children. RMS usually involves the extremities, the head and neck or the genitourinary system. Although it can arise from anywhere in the body, other sites of involvement are rare and usually present only at an advanced stage owing to a mass effect on surrounding tissues and organs. We present a rare case of a child who presented with the signs and symptoms of an acute abdomen, but intraoperatively was found to have a bleeding necrotic mass arising from the anterior abdominal wall. This was histologically confirmed to be a RMS of the embryonal type.

## Background

Around 6% of cancers in children and 1% of cancers in adults are attributed to soft tissue sarcomas. Half of sarcomas found in the paediatric age group are found to be rhabdomyosarcomas (RMS). RMS is a high-grade, malignant neoplasm in which cancer cells have a propensity for myogenic differentiation.^1^ RMS is divided into four groups: alveolar, embryonal, pleomorphic and spindle cell/sclerosing, with embryonal being the most common variant. This is based on the fourth edition of the World Health Organization Classification of Tumours of Soft Tissue and Bone.^2^ Whereas the alveolar variant usually involves extremities, the embryonal variant is most commonly noted to arise from the head and neck including the eye socket, and in genitourinary sites. Few comprehensive studies on the signs and symptoms of RMS in children exist.^1^ We report a case of RMS in a child who presented with an acute abdomen and was taken to theatre for presumed appendicitis but was found intraoperatively to have a bleeding necrotic mass arising from the anterior abdominal wall. Histopathological examination confirmed an RMS of the embryonal type.

**Figure 1:**
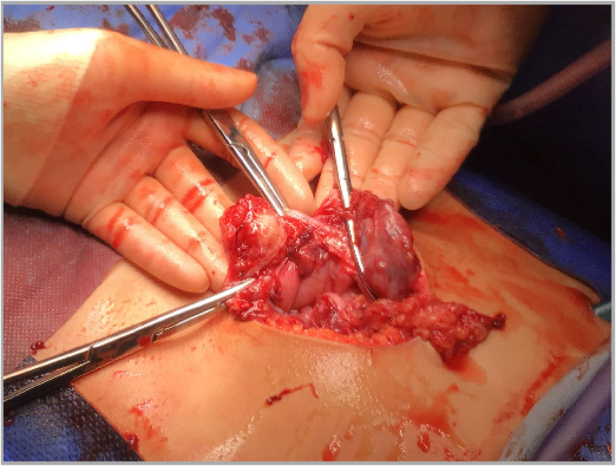
Intraoperative appearance of the mass

**Figure 2:**
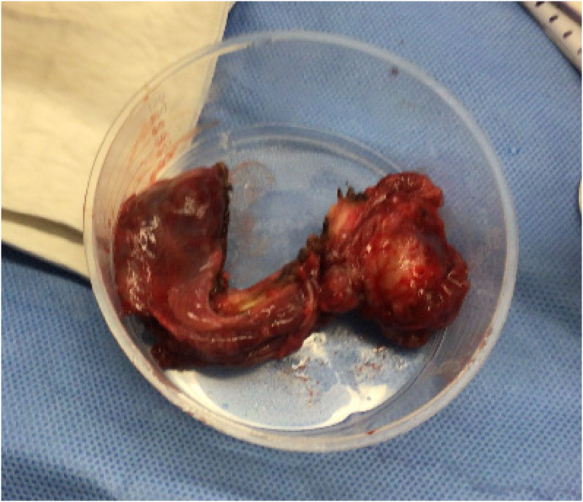
Mass after excision

## Case history

A 9-year-old boy presented to the paediatric emergency department with a 2-day history of lower abdominal pain that was sudden in onset, continuous in nature and aggravated on moving. He was asymptomatic prior to this. The pain began around the umbilical region and then migrated to the suprapubic and right iliac fossa (RIF) region. It was associated with vomiting, an episode of low-grade pyrexia, lethargy and anorexia. There was no history of trauma, recent respiratory/gastrointestinal symptoms, foreign travel or developmental/growth concerns. His mother commented that his older brother had presented a few years earlier with exactly the same symptoms and was found to have appendicitis.

Past medical history included anaemia as an infant, requiring treatment. There were no current comorbidities and no surgical history. There was no immediate family history of note but a maternal uncle had passed away from neuroblastoma.

On initial presentation, his heart rate was between 100 and 120bpm, respiratory rate 18 breaths/min and he had a temperature of 37.1°C. When called for examination, the patient was noted to be holding his right lower abdomen. There were no signs of anaemia or jaundice but he appeared dehydrated. Examination of his abdomen revealed tenderness in the suprapubic and RIF area with guarding.

Blood reports showed a slightly raised C-reactive protein of 18mg/L, a white cell count of 14.9 × 10^9^ cells/L with a neutrophil count of 12.07 × 10^9^ cells/L, haemoglobin of 113g/L and normal renal function. Urine analysis showed the presence of trace blood. The clinical impression was acute appendicitis. No preoperative imaging was undertaken because it was a weekend evening resulting in no access to a paediatric ultrasound.

On insertion of the first port and camera, fresh blood was seen in the peritoneal cavity. A necrotic haemorrhagic peritoneal mass measuring ∼4 × 3cm in diameter was found in the midline of the anterior abdominal wall below the umbilicus (Figure 1). Upon advice from the regional paediatric surgery unit, the case was converted to a laparotomy with an infraumbilical incision and the mass was resected to achieve control of the bleeding (Figure 2). Insertion of a urinary catheter suggested no attachment of the mass to the bladder. Intraoperatively the patient’s haemoglobin dropped to 79g/l warranting an urgent transfusion of blood during the operation. Estimated blood loss was 900ml. The rest of the laparotomy was unremarkable.

There were no concerns postoperatively and post-transfusion haemoglobin increased to 98g/l. The patient was discharged on day 3 after surgery.

Histopathological examination of the mass revealed it to be a high-grade soft tissue sarcoma with morphology and immunoprofiling consistent with RMS of the embryonal type with no high-risk gene fusion. In light of this information, the patient was referred to a specialist centre having paediatric oncology for further management.

## Discussion

In this case, the child was completely asymptomatic with no constitutional symptoms prior to the 48h period before presentation. He presented acutely with a 2-day history of symptoms. Compared with the case series by Gonébo *et al*, the case with the least number of days of evolution of symptoms was 14 days, whereas in the case report by Aljabban *et al*, it was noted that the patient presented with a 3-day history of symptoms. Intra-abdominal sarcomas tend to present late, as very large masses that can extend to the thoracic region. We believe this presentation as an acute abdomen resulting from a small but haemorrhagic mass is unusual for rhabdomyosarcomas.

In addition, all the cases in discussion in both the case series by Gonébo *et al* as well as the review of literature by Aljabban *et al* had a computed tomography (CT) scan performed for diagnostic purposes. However, this was not done in our case as a result of the acute presentation and signs and symptoms suggestive of acute appendicitis, and challenges within the hospital in obtaining paediatric imaging over weekends.^3,4^

Upon review by the paediatric oncology team, it was concluded that this acute presentation was as a result of likely spontaneous rupture of the mass. Postoperative CT and magnetic resonance imaging showed no residual mass. The patient also underwent positron emission tomography scanning, which showed no evidence of metabolically active disease elsewhere. But given the presumption of rupture and therefore spread, it was considered to be ‘very high risk’ and the patient was offered treatment based on the Far-RMS protocol, involving a combination of chemotherapy, radiotherapy and testicular cryopreservation.

## Conclusion

Subtle changes in this patient’s observations (tachycardia) led to urgent surgery prior to obtaining any intra-abdominal imaging. However, although this is a rare case, it has led to changes in paediatric radiology service provision in our trust (increased out-of-hours ultrasound as well as low-dose CT scans).
